# Miscellany

**Published:** 1906-08

**Authors:** 


### MISCELLANY.

A board of officers will be convened to meet at the Bureau of Public Health and Marine Hospital Service, 3 B Street, S. E., Washington, D. C., Monday, August 6, 1906, at 10 o'clock A. M., for the purpose of examining candidates for admission to the grade of assistant surgeon in the Public Health and Marine Hospital Service. For full information, or for invitation to appear before the board of examiners, address: The Surgeon-General, Public Health and Marine Hospital Service, Washington D. C.

The Morse International Agency of New York and London, one of the largest advertising houses in this country, is represented in Buffalo and the west by the Macdonald-Olmsted Advertising Company, which has opened commodious and well appointed offices in the German-American bank building, Buffalo. This is one of the finest locations in the city and bespeaks the good judgment of both principal and resident agencies.

